# Effects of three different bleaching agents on microhardness and roughness of composite sample surfaces finished with different polishing techniques

**DOI:** 10.4317/jced.53136

**Published:** 2017-03-01

**Authors:** İhsan Yikilgan, Hanife Kamak, Sinem Akgul, Suat Ozcan, Oya Bala

**Affiliations:** 1 Assist. Prof. Dr., DDS, PhD. Gazi University Faculty of Dentistry, Department of Restorative Dentistry, Ankara, Turkey; 2 Research Assistant. Gazi University Faculty of Dentistry, Department of Restorative Dentistry, Ankara, Turkey; 3 Prof. Dr., DDS, PhD. Gazi University Faculty of Dentistry, Department of Restorative Dentistry, Ankara, Turkey

## Abstract

**Background:**

The aim of this study was to evaluate effects of different polishing methods and whitening agents on surface hardness and roughness of nano-hybrid composite resin.

**Material and Methods:**

In total, one hundred twenty disc-shaped specimens were prepared to nano-hybrid composite (Charisma Diamond). 60 samples were used for microhardness measurements and the others were used for the evaluation of surface roughness. Samples were divided randomly into two subgroups (n = 30 each). In first group a low-viscosity liquid polishing agent (Biscover LV) was applied. In the second group, nothing was applied. All the samples were stored in distilled water at 37°C for 24 h. After initial measurements were completed, samples were divided randomly into three subgroups for bleaching application. 10% carbamide peroxide (Opalescence PF), 45% carbamide peroxide (Opalescence PF Quick), 38% hydrogen peroxide (Opalescence Boost) was applied. Then microhardness and surface roughness measurements of samples were repeated and data were recorded as final values for each sample.

**Results:**

When the polishing techniques were compared, no signicant difference was observed in surface hardness and roughness. When the bleaching agents were compared, the 10% carbamide peroxide and 38% hydrogen peroxide containing bleaching agent groups showed statistically significant differences between pre- and post-procedure hardness values (*p*<0.05).

**Conclusions:**

Office-type bleaching agent containing CP was observed to be more secure for composite resins than other bleaching agents. No negative effect of glaze materials on the protection of surface roughness and hardness of composite resin was observed.

** Key words:**Composite resin, bleaching, surface hardness, surface roughness.

## Introduction

Today, with an increase in esthetic expectations, esthetic applications have also gained in popularity. Bleaching is one non-invasive application that can protect natural dentition and meet esthetic expectations. Bleaching agents bleach the tooth by creating an oxidative reaction. There may be various negative effects of oxidative reactions on tooth tissues and restorative dental materials. Haywood *et al.* (1) reported that different concentrations of bleaching agents resulted in decreases in enamel microhardness. Also, these agents may cause increases in surface roughness and decreases in the surface hardness of restorative materials (2-6).

Surface roughness and hardness are important markers for the clinical success of restorations. Plaque accumulation, discoloration, gingival irritation, and secondary caries may be observed on rough restoration surfaces. Additionally, materials that have reduced surface hardness are more susceptible to deformation. Surface roughness and hardness of composite restorations are affected by structural properties of the material, such as monomer type, filler type, and percentage. Also, finishing and polishing restorations influence roughness and the hardness of composite materials. Finishing and polishing instruments and surface sealants are used to smooth restoration surfaces. Surface sealants are resin-based materials with high organic content, applied to restoration surfaces, to cover micro-pitting on restorations. The use of these materials, also discussed at the beginning of the 1990s, has been controversial (7). Although some researchers concluded that surface sealants had positive effects on the physical properties of restorations, others disagreed (8-13).

Many studies have evaluated the effects of bleaching processes on the surface roughness and hardness of composite resins. However, no reported research has evaluated the effects of bleaching processes on the surface characteristics of composite resins polished with a surface sealant.

The main aim of this study was to evaluate the changes in surface roughness and hardness of composite resin polished with a surface sealant. For this purpose, bleaching agents with three different compositions and concentrations were used (Opalescence PF, Opalescence PF Quick, and Opalescence Boost). Thus, we sought to determine the relationship between surface roughness and hardness of resin composites and the type of bleaching agent.

## Material and Methods

-Preparation of Samples

Materials used in this study and chemical components are shown in  Table 1. Composite resin (Charisma Diamond, Heraeus Kulzer, Germany) samples were prepared using 5-mm diameter and 2-mm deep Teflon molds. After composite resin was placed in the molds, a Mylar strip (Henry Schein, Melville, NY, USA) was placed, and a glass slab was laid on the mold. Then, the composite material was polymerized with a LED light curing unit (G Light, GC, USA) for 20 s according to the manufacturer’s protocol. Composite samples were polished with aluminum oxide-coated polishing discs (Sof-Lex, 3M ESPE, USA) 10 times for each disc on the surface of the samples. In total, 120 samples were prepared. Of them, 60 samples were used for microhardness measurements and the remaining samples were used for the evaluation of surface roughness.

**Table 1:**
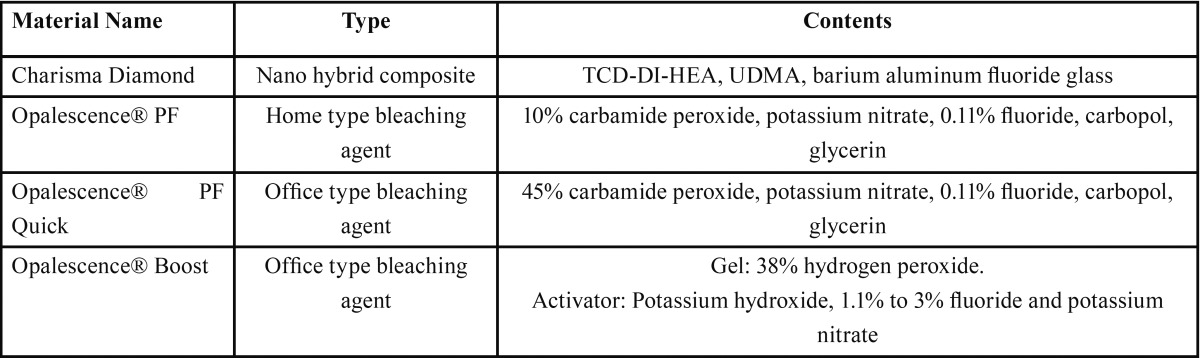
Materials used in this study.

Samples were divided randomly into two subgroups (n = 30 each). A low-viscosity liquid polishing agent, Biscover LV (Bisco, Schaumburg, IL, USA), was used with the first group of samples in accordance with the manufacturer’s instructions. First, 37% orthophosphoric acid was applied to the sample surface for 15 s, and then rinsed and dried with an air-water spray. A thin coat of Biscover LV was applied by brush on the sample surfaces, dried with mild air, and polymerized with a LED light curing unit for 30 s after waiting for 15 s for evaporation without air application. In the second group, nothing was applied to the surfaces as a control. All the samples were stored in distilled water at 37°C for 24 h.

-Microhardness measurements

Initial microhardness measurements were made with a Vickers hardness testing machine (Shimadzu HMV-2, Japan). Measurements were performed on samples with a 100-g load applied by the indenter for 30 s and this measurement was repeated in three different regions of each sample. The mean microhardness was calculated using the values of the three indentations and recorded as the Vickers hardness value of the sample.

After initial microhardness measurements were completed, composite samples in each group were divided randomly into three subgroups for bleaching application. Bleaching agent containing 10% carbamide peroxide (Opalescence PF; Ultradent Products Inc., South Jordan, UT, USA) was applied 8 h per day for 14 days, in accordance with the manufacturer’s instructions. Bleaching agent containing 45% carbamide peroxide (Opalescence PF Quick; Ultradent Products Inc.) was applied according to manufacturer’s instructions on surfaces of samples in Group 2 for 30 min and this application was repeated for three times. Bleaching agent containing 38% hydrogen peroxide (Opalescence Boost; Ultradent Products Inc.) was applied according to the manufacturer’s instructions on the sample surface in Group 3 for 20 min, twice, over 2 days. After the application of the bleaching agents, micro-hardness measurements of samples were repeated and data were recorded as final microhardness values for each sample.

-Surface roughness measurements

Initial surface roughness measurements of the samples were conducted using a profilometer roughness measuring device (SJ-301 Mitutuya Surfest, Japan). The end of the profilometer device was in contact with the centre of the sample, as far as possible, during the measurement, and it was performed from a distance of 0.8 mm, and repeated for three different regions for each sample. The mean of the three measurements was recorded as the surface roughness value of the sample. After the initial surface roughness measurement, composite samples in each group were divided randomly into three subgroups for bleaching application, as was done for the surface hardness samples. After application of the bleaching agent, surface roughness measurements of samples were repeated and data were recorded as the final surface roughness value for each sample.

-Statistical Analysis

Statistical analyses were performed using the SPSS software (ver. 15.0 for Windows). The normal distribution of the continuous quantitative variables was evaluated with the Kolmogorov-Smirnov test and the homogeneity of variance was evaluated with Levene’s test. As the data were normally distributed, descriptive statistics were used.

The significance of differences in mean values between groups was evaluated by one-way ANOVA. Bonferroni corrections were used with a post hoc test to control type 1 error in multiple comparisons. The importance of mean value differences within groups before and after the procedures was investigated with paired t-tests. *P* values < 0.05 were considered to indicate statistical significance.

## Results

Descriptive statistics of surface hardness results are shown in  Table 2. When the polishing techniques were compared, no significant effect was found between the groups in terms of protecting surface hardness (*p* > 0.05).

**Table 2:**
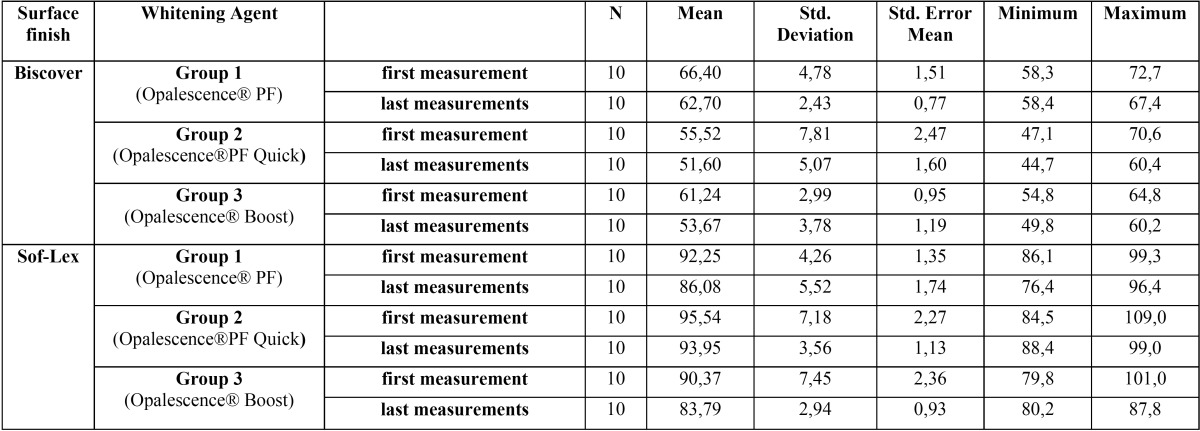
Descriptive statistics data of surface hardness results of the samples (mean, standard deviation, standard error mean, minimum and maximum values).

When the bleaching agents were compared, the 10% carbamide peroxide- and 38% hydrogen peroxide-containing bleaching agent groups showed statistically significant differences between pre- and post-procedure hardness values upon surface sealant application (*p* < 0.05;  Table 3).

**Table 3:**
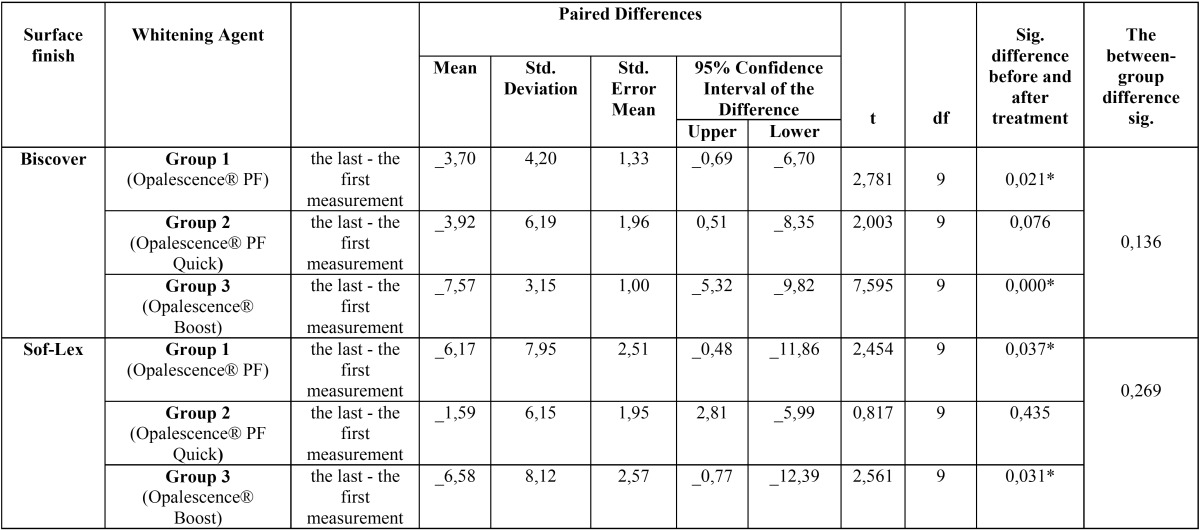
Statistical evaluation of the relationship between the amount of changes in surface hardness before and after the application of bleaching agent for all experimental groups.

Descriptive statistics of the surface roughness measurements shown in  Table 4. When different polishing techniques were compared, it was found that applying Biscover LV, or not, had no significant effect on surface roughness (*p* > 0.05). As a result of evaluating the bleaching agents, no statistically significant difference was found between the surface roughness measurements before and after bleaching in any group (*p* > 0.05;  Table 5).

**Table 4:**
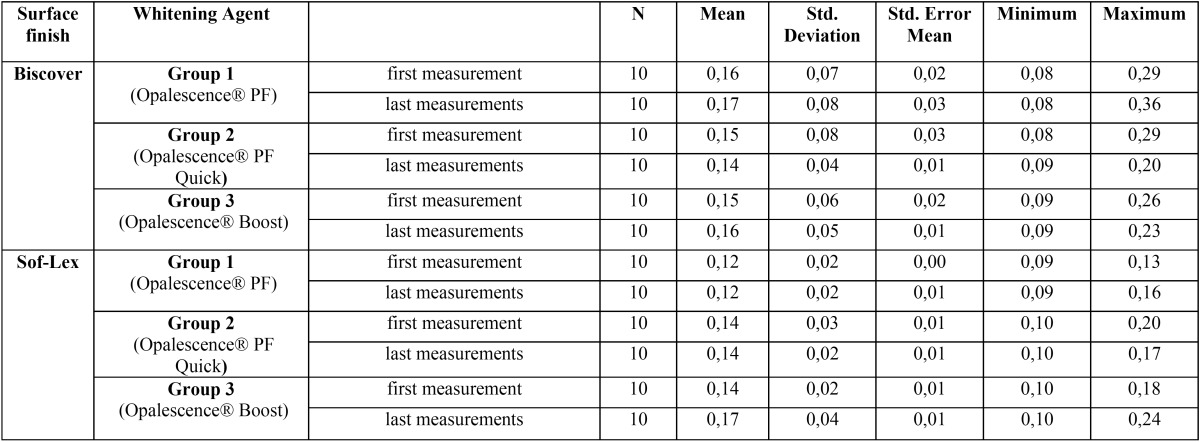
Descriptive statistical data of surface roughness of sample (mean, standard deviation, standard error mean, minimum and maximum value).

**Table 5:**
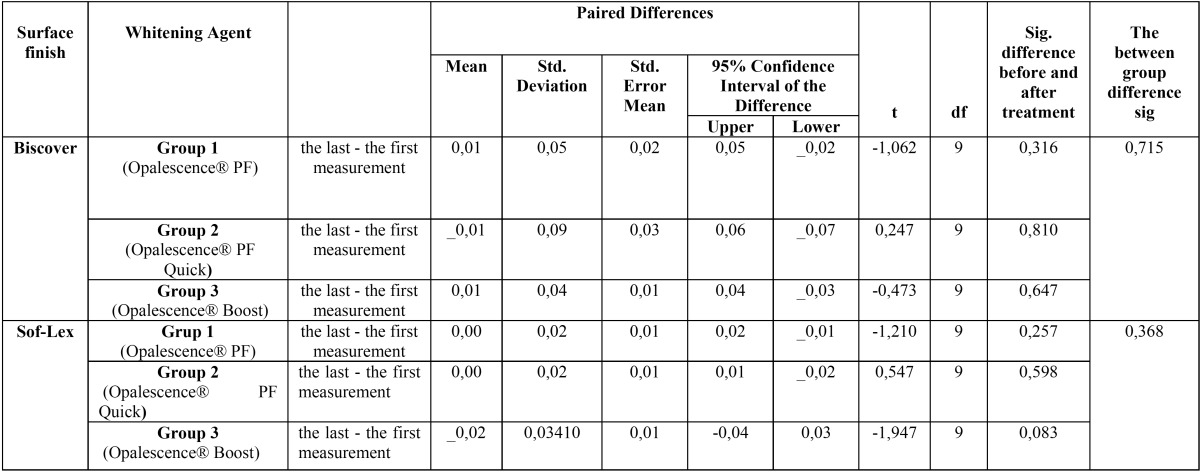
Statistical evaluation of the relationship between the amount of changes in surface roughness before and after the application of bleaching agent for all experimental groups.

## Discussion

Surface sealants are one of the alternatives that can be used for ensuring a smooth surface on composite restorations. Many studies have examined the effects of composite surface sealants on the properties of composite resin surfaces. However, there has been no previously reported study that evaluated how surface properties of composite resins are affected by bleaching agents after surface sealant application. Thus, our study is unique from this perspective.

Some researchers have reported effects of different aging applications on surface sealants using composite resins. Catelan *et al.* (11) applied artificial aging to composite resins under UV light, Briso *et al.* (14) applied aging to composite resins with different acidic solutions, and Karaaslan *et al.* (10) applied aging to composite resins with thermal cycling. In their results, surface roughness of the surface sealant samples used in all these studies was not affected adversely by the aging processes. In our study too, no significant difference was seen between the glazed and non-glazed samples in surface roughness changes. Thus, our results were consistent with these previous studies.

Many studies have evaluated the effects of bleaching agents on the surface roughness of composite resins. Some studies have reported that bleaching agents have no significant effects on the surface roughness of composite resins, while others have reported that bleaching agents increased the surface roughness (15-17). In our study, none of the bleaching agents tested showed any significant effect on the surface roughness of the composite resin. The roughness values of some samples in the experimental groups in our study were more than 0.2 µm. Generally, 0.2 µm, the average surface roughness of the experimental groups, is considered a clinically acceptable value.

Differing results have been reported in studies that have examined the effects of bleaching agents on the surface hardness of composite resins. Some studies have reported that the bleaching agents have no significant effect on the roughness of composite resins (18-20), while others have reported that they reduce the hardness of the surface (21-23). In our study, while office-type bleaching agent containing 38% HP and home bleaching agent containing 10% CP caused significant reductions in surface hardness of the composite resin, an office-type bleaching agent containing 45% CP did not cause any significant effect on the surface hardness of the composite.

Differences in office-type whitening agents may have resulted from the difference between the concentrations of agents. When CP is in contact with water, H202 emerges, in as much as a 30-40% concentration (24). That we used 45% CP gel that generates H202 to about half of that level in the gel containing HP in our study may have been why there was no significant change in surface hardness in the CP groups.

Differences between application periods may have affected the difference between home bleaching gel containing CP and office-type gel containing CP. While home bleaching gel was applied for 8 h every day for 14 days, office-type gel was applied three times for 30 min, according to the instructions. Although the office-type gel had a lower concentration, the long implementation period may have been more effective in damaging the surface of the composite resin. Malkondu *et al.* (23) evaluated the effects of two different home bleaching gels on surface hardness of different ceramic and composite resins. While the agent used for short-term application caused hardness changes in only one composite group, the long-term application agent caused deterioration of hardness in all the ceramic and composite groups. Lima *et al.* (22) evaluated the effects of home bleaching gel containing CP and office-type gel containing HP on the surface hardness of composite resins. They reported that the home bleaching agent caused a significant reduction in surface hardness of the composite resin compared with the control group. The results of these studies are consistent with our results.

Studies have reported that the use of glaze material reduces the surface hardness of composite resins but have also reported an increase in resistance to degradation in composite resins (11,14). In our study, it was seen that the initial surface hardness of groups where glaze material was used was significantly lower than the groups where glaze material was not used. However, it was found that bleaching applications had no significant effect on hardness. Thus, our work is consistent with these previous studies.

## Conclusions

1) Office-type bleaching agent containing CP was observed to be more secure for composite resins than an office-type bleaching agent containing HP.

2) Despite the low concentrations, home bleaching agents can lead to a significant decrease in the surface hardness of composite resins because of their long application period. Thus, for the bleaching process of teeth restored with composite resin, office-type bleaching agent containing CP that is applied for a shorter period of time may be more appropriate.

3) No effect of the bleaching agents on surface roughness of the composite resins was observed.

4) No negative effect of glaze materials on the protection of surface roughness and hardness of composite resin was observed.
